# Astrocytes in systemic regulation of blood glucose and the development of type 2 diabetes

**DOI:** 10.3389/fncel.2026.1850636

**Published:** 2026-07-01

**Authors:** Zhen Wen, Peng Bai, Li Du, Qianru Zhao, Mengliu Yang, Jialin Shan, Xiang Hu, Jie Min, Kangli Qiu, Yunfei Liao, Tianshu Zeng, Nan Zhang

**Affiliations:** 1 Department of Endocrinology, Union Hospital, Tongji Medical College, Huazhong University of Science and Technology, Wuhan, China; 2 Department of Cardiovascular Surgery, Union Hospital, Tongji Medical College, Huazhong University of Science and Technology, Wuhan, China; 3 Department of Gastroenterology, Union Hospital, Tongji Medical College, Huazhong University of Science and Technology, Wuhan, China; 4 Department of Biological Chemistry, School of Pharmaceutical Sciences, South-central Minzu University, Wuhan, China; 5 Department of Endocrinology, the Second Affiliated Hospital, Chongqing Medical University, Chongqing, China; 6 Institute of Hematology, Union Hospital, Tongji Medical College, Huazhong University of Science and Technology, Wuhan, China; 7 Diabetes and Metabolic Disease Clinical Research Center of Hubei Province, Wuhan, China; 8 Hubei Key Laboratory of Metabolic Abnormalities and Vascular Aging, Huazhong University of Science and Technology, Wuhan, China; 9 Hubei Branch of National Center for Clinical Medical Research of Metabolic Diseases, Huazhong University of Science and Technology, Wuhan, China

**Keywords:** astrocytes, glucose homeostasis, hypothalamus, neuron–glia interaction, type 2 diabetes

## Abstract

The central nervous system (CNS) relies primarily on glucose for energy, and therefore maintaining peripheral glucose homeostasis is essential to ensure adequate CNS energy supply and normal physiological functions. Historically, hypothalamic neurons—particularly defined glucose-sensing neuronal populations—were considered the main sensors and regulators of systemic glucose, while astrocytes primarily serving a nutritional and supportive role for neurons. However, recent studies have progressively revealed that astrocytes also play a significant role in systemic glucose regulation, particularly within the hypothalamus. In this review, we discuss the molecular mechanisms by which hypothalamic astrocytes sense glucose and modulate glucose homeostasis, and we examine how astrocyte–neuron interactions cooperate to maintain systemic glucose balance. We also summarize evidence that astrocytes in other regions, such as the hindbrain, participate in glucose regulation. Finally, we consider how altered astrocyte function may contribute to the development and progression of type 2 diabetes through changes in metabolism and inflammatory or signaling pathways. Research in this field not only expands our understanding of glial cell functions in metabolic regulation but also provides novel potential intervention targets for diseases associated with energy homeostasis imbalance, such as diabetes.

## Graphical abstract

**Figure:**
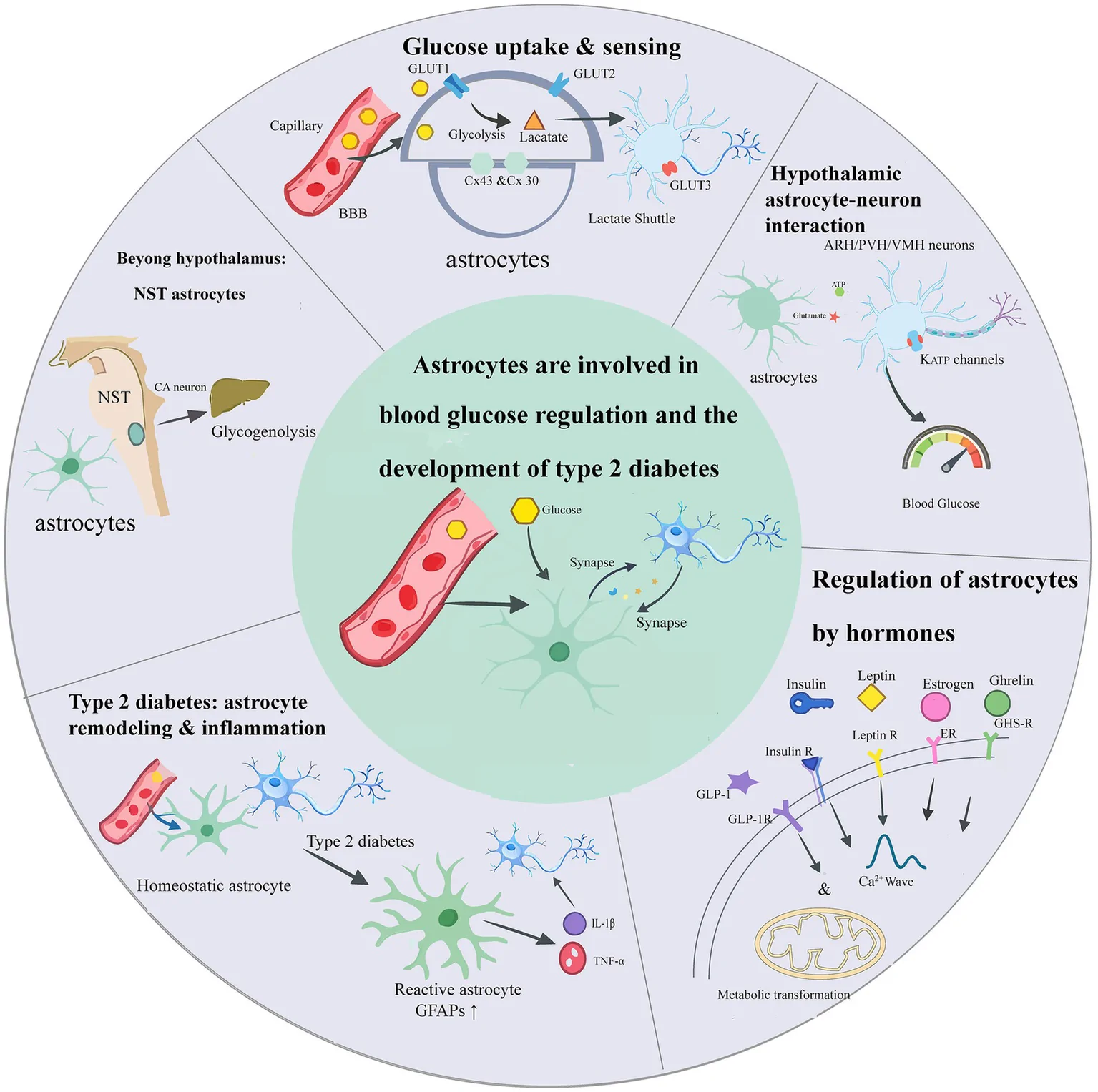
This review highlights the role of astrocytes in central glucose sensing and metabolic regulation. First, we summarize the mechanisms by which astrocytes sense fluctuations in glucose concentration, including glucose uptake via glucose transporters (GLUT1/GLUT2), intracellular metabolic processing (glycolysis and lactate production), as well as the interastrocytic network formed by Cx43 and Cx30. We then discuss how hypothalamic astrocytes interact with neurons in the arcuate nucleus of the hypothalamus (ARH), paraventricular nucleus of the hypothalamus (PVH), and ventromedial hypothalamus (VMH), through synaptic contacts and gliotransmitter release to regulate systemic glucose homeostasis. Notably, these astrocyte–neuron interactions are dynamically modulated by metabolic hormones, such as insulin, leptin, and GLP-1. Under pathological conditions such as type 2 diabetes (T2DM), astrocytes may undergo reactive astrogliosis, characterized by hypertrophy of cellular processes, and the release of pro-inflammatory mediators that act on neighboring neurons, thereby contributing to disease onset and progression. In addition to hypothalamic astrocytes, astrocytes in the nucleus of the solitary tract (NTS) in the medulla also interact with catecholaminergic (CA) neurons to regulate blood glucose levels in response to hypoglycemic stimuli. Together, these mechanisms highlight the critical role of astrocytes in central glucose sensing and metabolic regulation.

## 1 Introduction

The central nervous system precisely regulates food intake, energy expenditure, and glucose homeostasis by integrating peripheral metabolic substrates and hormonal signals. Among these, the hypothalamus serves as a critical hub for metabolic homeostasis, participating in process such as glucose and lipid metabolism (Lee et al., 2021), feeding behavior, and thermoregulation (Jolicoeur et al., 1995). Signals related to blood glucose fluctuations converge extensively in the mediobasal hypothalamus (MBH), primarily involving regions such as the dorsomedial nucleus (DMH), ventromedial nucleus (VMH), arcuate nucleus (ARH), and median eminence (ME). Glucose-sensing neurons are predominantly distributed in these brain regions. When blood glucose concentration rises, the firing rate of glucose-excited (GE) neurons increases, while glucose-inhibited (GI) neuron activity decreases. The opposite occurs during glucose depletion (Dunn-Meynell et al., 2002).

Beyond the hypothalamus, the hindbrain also serves as a critical node for central glucose sensing and systemic blood glucose regulation. The medulla-pons region contains metabolism-related nuclei such as the nucleus tractus solitarius (NTS) (Fozzato et al., 2023), the area postrema (AP) (Bruce et al., 2024), the dorsal vagal nucleus (DMV) (Takahashi et al., 2003), the dorsal raphe nucleus (DR) (Martin et al., 2023), and the parabrachial nucleus (PBN) (Lin et al., 2025). These nuclei form a distributed network for blood glucose regulation, interconnected with the hypothalamus through bidirectional connections. Glucose-sensing neurons are also present in the hindbrain. The hindbrain’s distinctiveness lies in the absence of a complete blood–brain barrier in certain structures (e.g., AP), allowing direct exposure to circulating metabolites and hormones. This makes it a crucial “window” for peripheral metabolic information to enter the CNS (Li et al., 2023). Furthermore, these regions are directly connected to peripheral organs—such as the liver, pancreas, and gut—via the vagus nerve, providing the anatomical foundation for rapid glucose regulation.

Based on the above findings, CNS-mediated blood glucose regulation has long been primarily explained through neuronal electrophysiological activity. However, recent research indicates a shift from this neuron-centric view, suggesting that CNS control of blood glucose likely involves a complex neuron–glia network. Glial cells play active roles as sensors-integrators-effectors within the metabolic information processing chain. Within this framework, astrocytes have garnered particular attention due to their numerical dominance and structural positioning. Astrocytes account for approximately 20–40% of brain and are particularly abundant in the hypothalamus (Khakh and Sofroniew, 2015). Traditionally, astrocytes were viewed primarily as structural and metabolic support cells for neurons, such as maintaining ion and neurotransmitter homeostasis (Almad and Maragakis, 2018), regulating synaptic activity (Baraibar et al., 2025), and participating in blood–brain barrier formation. However, advancing research has revealed that astrocytes are not passive “supporters” but can directly participate in and influence systemic glucose metabolism and energy balance (Chao et al., 2022). Morphologically, astrocytes possess highly branched stellate structures whose processes can simultaneously envelop presynaptic terminals, postsynaptic neurons, and blood vessels, forming the classic tripartite synapse configuration (Baldwin et al., 2024). This spatial arrangement allows astrocytes to establish extensive connections among neurons, blood vessels, and other glial cells, thereby finely regulating the local extracellular environment and intercellular signaling. More importantly, as a crucial component of the blood–brain barrier, astrocytes tightly cover capillary surfaces with their terminal processes (Kim et al., 2022), providing a unique anatomical advantage for directly sensing circulating metabolic substrates (such as glucose) and metabolic waste products. Additionally, astrocytes exhibit significant regional heterogeneity. Under basal condition, glycolytic activity in hypothalamic astrocytes adjusts primarily in response to glucose concentration changes. In contrast, cortical astrocytes exhibit a greater dependence on neuronal activity-related signals (such as glutamate) for their glycolytic response (Geller et al., 2025). This discrepancy suggests that the metabolic state of hypothalamic astrocytes can reflect glucose concentration fluctuations to a certain extent, providing new directions for understanding how glial cells sense blood glucose levels.

## 2 Astrocytes are essential gatekeepers of the central glucose sensing mechanism

In the CNS, astrocytes predominantly express Glucose transporter-1 (GLUT1), which supports basal glucose uptake. Additionally, in specific regions involved in metabolic sensing, such as the hypothalamus and brainstem, astrocytes also express Glucose transporter-2 (GLUT2), which plays a crucial role in glucose sensing. Together, these transporters facilitate both the basal utilization of glucose and the sensing of glucose levels in response to metabolic changes.

### 2.1 Glucose transporter-1

GLUT1, a high-affinity glucose transporter, is widely expressed in astrocytes and endothelial cells of the blood–brain barrier (BBB), where it serves as the principal mediator of glucose uptake (Simpson et al., 2007). Earlier localization studies have demonstrated that GLUT1 is present in gray matter astrocytes, particularly in regions close to blood vessels and synapses. This spatial distribution is highly suitable for its role as a glucose availability sensor (Morgello et al., 1995). Endothelial GLUT1 is critical for delivering peripheral glucose across the BBB into the brain. In astrocytes, GLUT1 is commonly regarded as the primary glucose transporter, responsible for the transport of glucose from the brain interstitium into the cell and serving as the prerequisite for cells to sense external glucose fluctuations. Within astrocytes, glucose undergoes glycolysis to form lactate, which is transported into neurons via monocarboxylate transporters (MCTs) before being converted to pyruvate (Pellerin and Magistretti, 1994). The remainder is converted into glycogen and stored within glial cells. Through this process, astrocytes link neural activity with local glucose utilization. Within this framework, the importance of GLUT1 lies in its role as the substrate entry point of this coupled pathway. Furthermore, Baraibar et al. (2025) found that specific knockout of GLUT1 in astrocytes blocks its response to glucose deprivation and eliminates the impact of glucose deprivation on synaptic transmission. Under glucose deprivation conditions, normal astrocytes exhibit enhanced Ca^2+^ signaling, a response that completely disappears in astrocyte-specific GLUT1 knockout mice. This Ca^2+^ response serves as a crucial initiating step for subsequent ATP release, adenosine production, and synaptic inhibition (Baraibar et al., 2025). This indicates that GLUT1-dependent glucose uptake indeed participates in astrocyte-mediated glucose sensing and synaptic regulation.

Interestingly, emerging evidence suggests that the role of astrocytic GLUT1 in systemic metabolism may be more complex than previously appreciated. Carlos G. Ardanaz et al. reported that reduced astrocytic GLUT1 actually improves central and peripheral glucose homeostasis (Ardanaz et al., 2024). Using hGFAP-Cre transgenic mice crossed with Slc2a1^flox/flox^ mice, they achieved conditional knockout of GLUT1 in CNS astrocytes starting from early development. In these GLUT1 deficient astrocytes, they observed enhanced insulin receptor-dependent ATP release, amplified hypothalamic insulin signaling, and activation of hypothalamic energy-sensing pathways, which in turn regulated systemic metabolism. In another study, Laetitia Thieren et al. employed the GLAST-CreERT2 inducible system and tamoxifen administration to adult mice to achieve specific knockout of GLUT1 in CNS astrocytes. They found that after the inducible deletion of astrocyte GLUT1 in adult mice, the mice’s sensorimotor and memory functions remained intact. Besides, astrocytes maintain normal resting glucose levels and exhibit enhanced glucose uptake and metabolic capacity unexpectedly (Thieren et al., 2025). Together, these findings reveal a reprogramming mechanism in astrocyte-neuron metabolic coupling, indicating that GLUT1 is not an absolute limiting factor for glucose uptake and homeostasis under all conditions. Its absence can trigger adaptive metabolic reorganization. This remarkable plasticity in astrocytes offers novel therapeutic insights for metabolic disorders.

### 2.2 Glucose transporter-2

In contrast, GLUT2 is a low-affinity, high-capacity glucose transporter expressed at lower levels and in a more region-specific manner, particularly in hypothalamic and brainstem areas involved in systemic glucose homeostasis (Pasula et al., 2024). Owing to its high Km for glucose, GLUT2 is not optimized for basal glucose uptake but is well suited for sensing fluctuations in extracellular glucose concentrations, thereby enabling GLUT2-expressing cells to function as glucose sensors.

During hypoglycemia, glucose sensors in peripheral tissues and the brain detect glucose deficiency and initiate a glucose regulatory response to restore glucose to normal levels, known as the counterregulatory response (CRR) (Cryer, 1999). Within this framework, astrocytes are increasingly recognized as contributors to central glucose sensing and CRR regulation (Rogers et al., 2020). Astrocytes expressing GLUT2 act as a sensor stimulating glucagon secretion during hypoglycemia. Mechanistically, Rogers and colleagues demonstrated that astrocytic GLUT2 engages phospholipase C (PLC)-dependent signaling, leading to calcium release from the endoplasmic reticulum. This intracellular calcium signaling modulates catecholaminergic neuronal output in the hindbrain, triggering CRR. This calcium response appears to rely primarily on intracellular stores rather than extracellular calcium influx (Rogers et al., 2020). In GLUT2 knockout mice, transgenic re-expression of GLUT2 restored glucagon responsiveness to hypoglycemia, suggesting that GLUT2 not only senses glucose concentration but also regulates blood glucose by modulating hormone sensitivity (Marty et al., 2005).

Notably, emerging evidence indicates that GLUT2 may interact with astrocytic metabolic sensors such as glucokinase (GCK), exhibiting sex-dependent regulatory effects. Specifically, GLUT2 has been reported to modulate GCK expression in a glucose-dependent manner in males, whereas this regulatory mechanism appears to be absent or attenuated in females, highlighting a potential sex dimorphism in hypothalamic astrocyte glucose sensing (Nampoothiri et al., 2022).

### 2.3 Glucose transporter-3

GLUT3, as the second most common transporter in the brain, is the primary glucose transporter in neurons (Peng et al., 2021). Its sequence shares 64% similarity with GLUT1 (Nguyen et al., 2021). The cDNA encoding mouse brain GLUT3 was isolated and sequenced by Nagamatsu et al. (1992) and the results were consistent with its role as a neuronal glucose transporter. Compared with other glucose transporters such as GLUT1 and GLUT2, Liang et al. elucidated Michaelis–Menten characteristics of GLUT3, low *K_M_* and high *V_max_*, indicating that GLUT3 possesses a higher glucose affinity and transport capacity, enabling it to effectively sustain neuronal energy supply even under hypoglycemic conditions (Liang et al., 2018). Studies have shown that half of the normal GLUT3 level is sufficient to meet the glucose demand of neurons (Stuart et al., 2011).

In addition to being present on neurons, GLUT3 is also present in astrocytes (Iwabuchi and Kawahara, 2011). However, current research mainly focuses on alterations of astrocytic GLUT3 under pathological conditions. Specifically, in the middle cerebral artery occlusion (MCAO) model, Gutiérrez Aguilar et al. found that GLUT3 expression was increased in astrocytes of the ipsilateral cerebral cortex, while it was also elevated in other cell types, including damaged neurons and microglia (Gutiérrez Aguilar et al., 2020). Similarly, following ischemia in the hippocampal CA1 region, GLUT3 immunoreactivity was markedly enhanced in astrocytes, peaking at seven days post-ischemia (Yoo et al., 2016). These results suggest that under conditions of restricted energy supply (such as local hypoglycemia or metabolic stress induced by ischemia), astrocytes may upregulate GLUT3 to enhance glucose uptake capacity, thereby coping with energy demands or supporting neuronal survival. However, because studies on glucose sensing by astrocytic GLUT3 remain limited and fall outside the glucose-sensing framework of this review, GLUT3 is discussed only briefly in this review.

### 2.4 Lactic acid

The concept of metabolic coupling between astrocytes and neurons via lactate exchange has emerged as a candidate mechanism underlying hypothalamic glucose sensing (Geller et al., 2025). In the hypothalamus, astrocytes and neurons achieve metabolic coupling through lactate shuttling, which not only serves as an energy substrate pathway but also a key mechanism for glucose sensing. Geller et al. demonstrated that cortical astrocytes follow a process termed the astrocyte-neuron lactate shuttle (ANLS), in which glutamate enhances glycolysis (Pellerin et al., 1998). Glutamate released into the synaptic cleft during neuronal activity is taken up by astrocytes, activating the Na+/K + -ATPase pump within astrocytes and thereby enhancing astrocytic glycolysis. The L-lactic acid produced by glycolysis is released into the extracellular space via monocarboxylate transporters (MCTs) and subsequently taken up by neurons as an energy substrate.

However, it is important to note that, unlike the mechanism described above, hypothalamic astrocytes can produce lactate directly in response to changes in glucose availability, independently of synaptic glutamate signaling. Hypothalamic astrocytes regulate lactate production in an AMPK (AMP-activated protein kinase)-dependent manner based on glucose concentration (rather than glutamate), establishing a glucose concentration-AMPK-lactate regulatory axis that serves as the core molecular basis for glucose sensing in these cells (Geller et al., 2025). Lactate release from hypothalamic astrocytes is highly sensitive to changes in glucose concentration, increasing in parallel with rising glucose levels. This sensitivity suggests that hypothalamic astrocytes possess the capacity for glucose sensing, with lactate serving as their output signal. By releasing lactate, astrocytes regulate neuronal excitability through pyruvate metabolism and activation of K-ATP channels, thereby participating in hypothalamic glucose sensing (Karagiannis et al., 2021).

### 2.5 Connexin

Connexin (Cx) is highly conserved across species and encoded by approximately 20 genes. Six connexin subunits collectively form a connexin hemichannel, and docking of two hemichannels from adjacent cells forms a gap junction channel (Scott et al., 2012). In the adult brain, Cx43 and Cx30 are the predominant Cx isoforms expressed in astrocytes (Nagy et al., 1999). They enable astrocytes to be extensively coupled into coordinated networks via gap junctions. Glucose that enters a single astrocyte can diffuse throughout the astroglial network via Cxs, thereby providing energetic fuel to neurons, which is particularly important for relaying metabolic information to glucose-sensing neurons that are not located in the immediate vicinity of blood vessels (Giaume et al., 2010). This network architecture thus provides a critical pathway for the intercellular exchange of ions and metabolites such as glucose and lactate (Harris, 2007).

Going a step further, this astroglial gap junction network has been demonstrated to be essential for hypothalamic glucose sensing (Allard et al., 2014). Camille Allard et al. employed RNA interference to inhibit Cx43 expression in the MBH and found that the insulin secretory response to elevated blood glucose was significantly attenuated in rats. This result directly indicates that the Cx43-constituted connexin network is required for the central glucose-sensing mechanism (Allard et al., 2014). Notably, when RNA interference led to a reduction in Cx43 protein levels, Cx30, which co-forms gap junction channels with Cx43, was also decreased, suggesting that the loss of Cx43 may indirectly affect Cx30 expression (Allard et al., 2014). In addition, fluctuations in blood glucose can directly modulate the expression of astroglial connexins. Cx43 levels decrease significantly under fasting conditions, whereas Cx43 expression increases rapidly during hyperglycemia (Allard et al., 2014). This pattern is consistent with its function in mediating the intercellular and intrahypothalamic trafficking of glucose and its metabolites (e.g., lactate), suggesting that this adaptive regulation of gap junction coupling may support coordinated metabolic responses within the hypothalamus.

Regarding Cx26, although Nagy et al., using confocal immunofluorescence double-labeling and FRIL (Freeze-Fracture Replica Immunogold Labeling), demonstrated that Cx26 is highly colocalized with the two known astrocytic gap junction proteins Cx30 and Cx43, and is directly present in astrocyte-to-astrocyte gap junctions (Nagy et al., 2001), evidence supporting a specific role for Cx26 in glucose sensing remains limited. Ball et al. cultured astrocytes in high-glucose (25 mmol/L) or low-glucose (5.5 mmol/L) medium for a prolonged period and found that, while the high-glucose environment led to an approximately 30% reduction in Cx30 protein levels and a 1.9-fold increase in Cx43 protein levels, the protein level of Cx26 did not change significantly (Ball et al., 2011). This result suggests that Cx26 is insensitive to changes in extracellular glucose concentration and is unlikely to be involved in astrocytic sensing and response to glucose levels. Therefore, this review will briefly discuss Cx26, with a primary focus on Cx30 and Cx43.

## 3 Astrocytes collaborate with neurons in various regions to regulate blood glucose levels

After explaining the mechanism by which hypothalamic astrocytes perceive glucose fluctuations, a critical question arises: how is this “metabolic sensing” translated into the regulation of systemic blood glucose homeostasis? Astrocytes do not act alone in response to energy signals but occupy a pivotal position at the junction between hypothalamic neural circuits and peripheral metabolism. Through tight structural coupling and functional interactions with neighboring neurons, astrocytes sense local glucose fluctuations, modulating neuronal excitability and synaptic transmission. This, in turn, influences downstream autonomic neural output and systemic blood glucose regulation.

Early studies have suggested a critical collaborative relationship between astrocytes and neurons. Young et al. disrupted astrocytic carbohydrate and glutamate metabolism in rats by injecting saline or methionine sulfonamide (MS). They found that neurons in the AP failed to detect 2-DG-induced hypoglycemic signals (Young et al., 2000). This suggests astrocytes likely occupy an upstream position in metabolic sensing circuits, acting as signal pre-processors. Furthermore, astrocytes can translate incoming metabolic information into alterations in neuronal function (Chowen et al., 2019), thereby influencing hypothalamic circuits. At the circuit level, multiple hypothalamic regions contribute to regulating energy homeostasis and glucose metabolism, including the ARH, VMH, DMH, paraventricular nucleus (PVN), and lateral hypothalamic area (LHA). Within these regions reside specific neuronal populations that rigorously monitor peripheral metabolic states. These populations influence food intake and body metabolism and possess structural pathways connecting to peripheral effector organs. For example, Rosario et al. used pseudorabies virus (PRV) retrograde tracing to demonstrate for the first time that the ARH, VMH, and LHA project directly to pancreatic islets (Rosario et al., 2016). This suggests that hypothalamic neurons can directly regulate insulin and glucagon secretion via neural pathways, rather than solely through indirect hormonal or metabolic routes. This establishes a clearer pathway: following the perception and “preprocessing” of metabolic signals by astrocytes, they influence the excitability and synaptic transmission of neurons within key hypothalamic nuclei (such as the ARH). These nuclei send direct neuronal projections to pancreatic islets to regulate peripheral hormone secretion and blood glucose homeostasis.

### 3.1 Arcuate nucleus

The arcuate nucleus (ARH) is a central hub housing distinct neuronal subpopulations with specialized functions, including AgRP/NPY-expressing neurons, proopiomelanocortin (POMC)-expressing neurons, dopaminergic neurons, GABAergic neurons, glutamatergic neurons, aromatase neurons, and so on. Currently, the primary focus of research in the ARH is on the neuropeptide Y/agouti-related peptide (NPY/AgRP) and pro-opiomelanocortin (POMC) neurons (Cone et al., 2001). Beyond regulating feeding behavior, POMC neurons also play a critical role in glucose homeostasis. Their intact function is particularly essential for normal counter-regulatory responses (CRR) to hypoglycemia (Tooke et al., 2019). Astrocytes within the ARH participate in central glucose sensing and regulation of peripheral blood glucose through tight functional coupling with AgRP/NPY and POMC neurons. Naiyan Chen et al. used electrophysiological and chemogenetic approaches to demonstrate that selectively elevation of Ca^2+^ signaling in ARH astrocytes leads to the release of certain chemicals, which significantly increases AgRP/NPY neuronal firing activity, thereby promoting feeding accompanied by elevated blood glucose (Chen et al., 2016). However, the identity of these chemicals remains to be elucidated. Based on recent advances, these “chemicals” released by astrocytes are likely to be multiple signaling molecules such as adenosine (Wu et al., 2017), prostaglandin E2 (Varela et al., 2021), or endogenous peptides (Bouyakdan et al., 2019). Studies have demonstrated that adenosine, a degradation product of ATP released by astrocytes, modulates AgRP neuron activity via adenosine A₁ receptors, influencing feeding behavior and glucose homeostasis (Yang et al., 2015). Under hunger or metabolic stress, GABA released by AgRP neurons activates astrocytes. This process induces GFAP upregulation and mitochondrial morphological alterations in ARH astrocytes. Concurrently, it increases astrocytic ensheathment of AgRP cell bodies and reduces inhibitory synaptic inputs to these cell bodies, thereby enhancing neuronal excitability. Simultaneously, GABA-activated astrocytes secrete PGE₂, forming a positive feedback loop that amplifies AgRP neuronal activity. Although validated through feeding phenotypes, this mechanism suggests that during fasting astrocytes may coordinate glucose homeostasis by amplifying AgRP circuit activity (Varela et al., 2021).

Additionally, astrocytes regulate neuronal activity by modulating energy substrate supply patterns. Specific knockout of the FoxO1 transcription factor in astrocytes shifts their metabolism from glycolysis to oxidative phosphorylation, reducing lactate production. This leads to excessive activation of NPY neurons in the ARH, exacerbating insulin resistance and resulting in systemic glucose dysregulation (Doan et al., 2023). Yoon et al.’s research further supports this: at physiological glucose concentrations, lactate enhances POMC neuronal discharge activity, with an activation effect significantly stronger than glucose itself. Mechanistically, lactate elevates the cytoplasmic NADH/NAD^+^ ratio via lactate dehydrogenase-mediated redox reactions, thereby activating mitochondrial oxidative phosphorylation and enhancing POMC neuronal excitability. This process relies on the intact function of uncoupling protein 2 (UCP2) in POMC neurons. Knockout of UCP2 in the POMC neurons significantly impairs their sensitivity to lactate and glucose, inhibits NADH production, and ultimately leads to impaired glucose tolerance and insulin resistance. These findings suggest that an astrocyte lactate–POMC neuron–UCP2 functional pathway plays a pivotal role in central glucose sensing and the regulation of systemic glycemic homeostasis (Yoon et al., 2022). Future studies should further investigate astrocyte–ARH neuronal coordination, focusing on neurotransmitter signaling and astrocytic glucose metabolism.

Notably, in addition to AgRP/NPY neurons and POMC neurons, other neuronal subpopulations in the ARH also contribute to the regulation of energy metabolism. Recent single-cell sequencing has identified a distinct ARH neuronal population expressing cellular retinoic acid–binding protein 1 (CRABP1). Although its physiological role remains unclear, ablation of CRABP1-expressing arcuate neurons (ARC^CRABP1^ neurons) leads to obesity and a diabetic phenotype in mice. Moreover, these neurons project to the PVH, NTS, and PBN, thereby modulating feeding behavior (Yan et al., 2025). Exploring potential interactions between astrocytes and ARC^CRABP1^ neurons will be an important direction for future research and may offer new insights into how the ARH influences metabolic homeostasis.

### 3.2 The paraventricular nucleus

Research has revealed that the paraventricular nucleus (PVN) of the hypothalamus is an important central hub that integrates autonomic nervous system outputs, regulating peripheral glucose metabolism through both sympathetic and parasympathetic. Preautonomic neurons within the PVN project to autonomic relay structures such as the rostral ventrolateral medulla (RVLM) and the intermediolateral cell column (IML) of the spinal cord, thereby influencing sympathetic and parasympathetic tone in peripheral organs and influencing metabolic homeostasis (Savić et al., 2022). With respect to the liver, enhanced neuronal activity in the PVN-liver pathway under diabetic conditions, potentially drives up sympathetic output and promoting hepatic glucose release, thereby contributing to the hyperglycemic phenotype (Gao et al., 2017). In contrast, parasympathetic pathways tend to reduce glucose production and promote glucose storage. Beyond influencing hepatic glucose production, the PVN also participates in glucose homeostasis by regulating insulin secretion. Papazoglou et al. identified a brain-to-*β*-cell multisynaptic pathway originating from a PVN oxytocinergic neuron subset (PVN^OXT^): activation of PVN^OXT^ neurons rapidly suppresses insulin secretion and elevates blood glucose; conversely, silencing these neurons prevents normal fasting-induced insulin suppression, resulting in marked hypoglycemia (Papazoglou et al., 2022). Within this PVN-peripheral organ regulatory axis, emerging evidence suggests that PVN astrocytes serve as critical regulators for PVN output. Herrera Moro Chao et al. demonstrated that obesity induces nucleus-specific remodeling of hypothalamic astrocytic Ca^2+^ activity. In the PVN, chemogenetic upregulation or downregulation of astrocytic Ca^2+^ signaling has been shown to bidirectionally modulate adjacent PVN neuronal firing, autonomic outflow, and systemic glucose metabolism and energy balance (Chao et al., 2022). Mechanistically, glia–neuron coupling depends on astrocytic clearance of extracellular glutamate (ambient glutamate) via excitatory amino acid transporters (EAATs), thereby maintaining the baseline excitability of PVN neurons. However, this mechanism is selectively impaired in the obese state. In obese mouse PVNs, reduced astrocytic glutamate transport (particularly GLT-1) impairs glutamate clearance, elevates extracellular glutamate, and disrupts glia–neuron homeostasis. Simultaneously, blockade of NMDARs decreases astrocytes’ regulatory capacity in controlling PVN neuronal firing, suggesting this process primarily modulates PVN network excitability via glutamate-NMDAR signaling mechanisms. Together, these findings support the concept that PVN astrocytes regulate the activity of pre-autonomic neurons by modulating extracellular glutamate–NMDAR signaling, thereby influencing sympathetic outflow and coordinating key metabolic processes, including hepatic glucose production and insulin secretion, ultimately contributing to obesity-associated impairments in glucose homeostasis.

### 3.3 Ventromedial hypothalamic nucleus

The ventromedial hypothalamic nucleus (VMH) is not a functionally homogeneous center for blood glucose regulation, but rather a highly heterogeneous region with distinct anatomical structure and molecular markers. It comprises multiple subregions including the dorsomedial (VMHdm), central (VMHc), and ventrolateral (VMHvl) of the VMH (López-González et al., 2023). Previous studies indicate that manipulating specific neuronal subpopulations within the VMH can significantly alter systemic blood glucose levels, glucose tolerance, and peripheral insulin sensitivity. Based on functional output, neuronal activity within the VMH can be broadly classified into two core tasks: First, glucose elevation and counter-regulatory response, primarily mediated by glucose-inhibitory (GI) neurons. These promote hepatic glucose output and release of glucagon and epinephrine via GABA/NO signaling pathways to counteract hypoglycemia. Second, hypoglycemic responses and glucose tolerance improvement, often involve some glucose excitatory (GE) neurons and glutamatergic outputs. These neurons inhibit hepatic glucose production and enhance peripheral glucose utilization via autonomic pathways (Tu et al., 2022). Within this intricate neural regulatory network, glial-neuronal interactions at the cellular metabolic level play a crucial upstream role. In the classical metabolic coupling model, astrocytes convert glucose into the oxidative fuel L-lactic acid via glycolysis, providing critical energy support for VMH neurons. Under pathological conditions, this metabolic support may undergo adaptive remodeling. In models of recurrent hypoglycemia and diabetes, enhanced lactate metabolism and transport within the VMH increase ATP production and close K-ATP channels. This process leads to an abnormal promotion of GABA release, inhibiting the counter-regulatory activation of neuronal activity. Consequently, glucagon and epinephrine fail to activate during hypoglycemia, leading to a breakdown in the body’s defense mechanisms against low blood sugar (Chan et al., 2013). Furthermore, recent studies have revealed the unique role of endogenous glucose production within astrocytes. Briski et al. discovered that VMH astrocytes specifically express glucose-6-phosphatase-*β* (Glc-6-Pase-β), enabling them to convert glycogen breakdown products into free glucose. This endogenous glucose production not only serves as an energy supply but also acts as a metabolic signal differentially regulating neurotransmission in the VMHdm and VMHvl. Inhibiting this enzyme alters GABA and nitric oxide synthase (NOS) levels, thereby weakening the body’s corticosterone and growth hormone secretion responses during hypoglycemia. This demonstrates that astrocytes function not only as energy factories but also as precise regulators of VMH’s integrity in counter-regulatory responses to hypoglycemia through dual metabolic pathways of lactate and glucose (Briski et al., 2025).

Beyond metabolic support, astrocytes directly modulate VMH neuronal firing activity by regulating neurotransmitter levels within the synaptic microenvironment. Regarding excitatory transmission, Meng et al. revealed the critical role of the metabotropic glutamate receptor 5 (mGluR5) in VMH astrocytes (Meng et al., 2023). This study revealed that mGluR5 on astrocytes modulates excitatory synaptic drive to VMH pituitary adenylate cyclase-activating polypeptide (PACAP^+^) neurons. Specific knockout of mGluR5 in VMH astrocytes reduced excitatory synaptic drive and synaptic input to the VMH (particularly PACAP^+^ neurons), attenuating PACAP neuron activity during acute hyperglycemia. This relieved the inhibitory effect on insulin secretion, manifesting as improved glucose tolerance and enhanced insulin secretion in response to high glucose stimulation. This indicates that under acute glucose stimulation, VMH astrocytic mGluR5 maintains excitatory synaptic input and responsiveness in VMH neurons (particularly PACAP^+^ neurons). This astrocyte-neuron cooperative mechanism provides central inhibitory regulation of glucose-stimulated insulin secretion (GSIS), thereby helping prevent excessive glucose clearance and reducing hypoglycemia risk.

## 4 Astrocytes are regulated by signaling molecules

**Figure 1:**
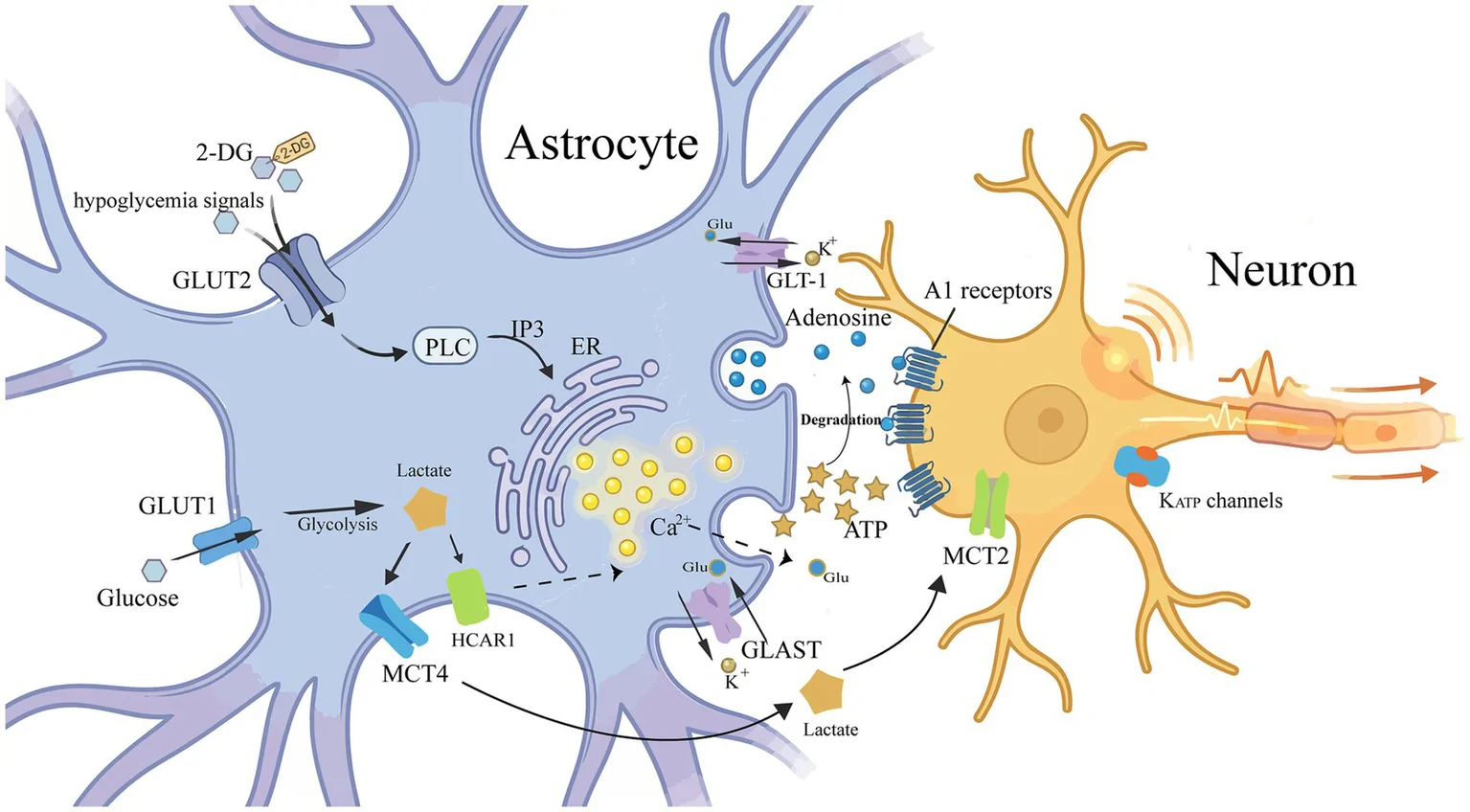
Mechanisms of astrocyte–neuron interaction. Astrocytes detect hypoglycemic signals induced by 2-DG via GLUT2 and activate phospholipase C (PLC) signaling to trigger Ca^2+^ store release from endoplasmic reticulum (ER), thereby elevating cytosolic Ca^2+^ levels. Elevated intracellular Ca^2+^ promotes the release of ATP, which is subsequently converted to adenosine, activating adenosine A1 receptors (A1Rs) on neurons and thereby influencing neuronal firing. Besides, the expression of GLT-1 and GLAST on NST astrocytes enhances glutamate clearance capacity, thereby maintaining extracellular glutamate homeostasis and indirectly regulating neuronal excitability. Metabolically, astrocytes take up glucose via GLUT1 and produce lactate intracellularly through glycolysis. Lactate can regulate neuronal activity through two pathways: (1) Lactate mediates activation of Hydroxycarboxylic Acid Receptor 1 (HCAR1), leading to increased intracellular calcium and subsequent glutamate release to neurons. The dashed line indicates a potential mechanism that has not yet been evaluated. (2) Lactate is transported out of astrocytes via Monocarboxylate Transporter 4 (MCT4) and taken up by neurons via MCT2 as an energy substrate. The increase in the ATP/ADP ratio causes the closure of ATP-sensitive K^+^ channels, producing a depolarization.

Hypothalamic astrocytes form close functional synergies with neurons through metabolic coupling and signaling processes associated with this coupling, such as glia–neuron communication mediated by molecules such as glutamate and ATP. Together, they jointly participate in the central regulation of blood glucose homeostasis (Figure 1). It is important to note that this glia–neuron synergy is not static but dynamically adjusted under the influence of peripheral endocrine signals. Hypothalamic astrocytes express multiple hormone receptors associated with metabolic regulation, enabling peripheral hormones to act directly upon astrocytes. This interaction leads to changes in astrocyte morphology, glucose processing capacity, release of signaling molecules like adenosine, and the efficiency of information exchange with neurons. Ultimately, this influences the hypothalamic neural circuit’s integration and its energy status output, contributing to the regulation of systemic glucose homeostasis. Therefore, the following sections will further explore how hormones regulate hypothalamic astrocytes to influence their synergistic interactions with neurons, thereby participating in the regulation of systemic glucose homeostasis.

### 4.1 Insulin

Insulin is a polypeptide hormone synthesized and secreted by pancreatic *β*-cells. Although the brain was traditionally considered an insulin-independent organ, modern research reveals widespread expression of insulin receptors throughout the CNS (Shaughness et al., 2020), suggesting insulin directly participates in central energy and metabolic regulation. Astrocytes, as the primary insulin target cells in the brain, are crucial for maintaining central metabolic balance. Correspondingly, the understanding of insulin action in the hypothalamus has evolved from a model where insulin primarily affects neurons to a more complex glia–neuron network regulation mediated by astrocytes, influencing glucose sensing and metabolism (Könner et al., 2007). At the molecular level, studies reveal that insulin signaling in hypothalamic astrocytes synergizes with insulin-like growth factor I (IGF-I) to promote translocation of GLUT1 from the cytoplasm to the cell membrane. This enhances astrocytic glucose uptake capacity, enabling more efficient neuronal energy supply (Fernandez et al., 2017).

Consistent with this, insulin also promotes a shift in astrocytic energy utilization from glucose toward fatty acid oxidation (Taib et al., 2025). Insulin rapidly upregulates the gene expression of pyruvate dehydrogenase kinase 4 (Pdk4) in astrocytes, thereby inhibiting the entry of glucose into the TCA cycle. Meanwhile, the expression of carnitine palmitoyltransferase 1a (CPT1a) is increased, which facilitates the entry of fatty acids into the TCA cycle and supplies energy to astrocytes through fatty acid metabolism. In this manner, more glucose can be spared and diverted to pyruvate for conversion into lactate that is delivered to neurons as their preferred fuel (Taib et al., 2025). This phenomenon is functionally complementary to the ANLS model. Notably, this metabolic switch between glucose and fatty acids is not exclusive to astrocytes, but rather represents a distinctive feature of hypothalamic nuclei in the regulation of energy balance. In the hypothalamus, the metabolism of glucose and long-chain fatty acids is tightly coupled. Elevated glucose concentrations inhibit AMP-activated protein kinase (AMPK) activity, which in turn weakens AMPK-mediated phosphorylation and inhibition of acetyl-CoA carboxylase (ACC), leading to increased ACC activity and enhanced malonyl-CoA production. As a potent inhibitor of carnitine palmitoyltransferase-1a (CPT-1a), malonyl-CoA effectively restricts the entry of long-chain fatty acids into mitochondria for *β*-oxidation (Taïb et al., 2013). This glucose–fatty acid metabolic coupling mechanism provides a different perspective for understanding the role of insulin in astrocytes.

In addition to regulating substrate allocation, astrocytic insulin signaling also plays a role in the morphological and functional coupling between glial cells and neurons. Research by Garcia-Caceres et al. revealed that mice with specific knockout of astrocytic IR under hyperglycemic exposure exhibited remodeling and altered activity in hypothalamic astrocytes and neurons. This suggests that astrocytic insulin signaling is involved in regulating the morphology and function of the tripartite synapse between astrocytes, neurons, and blood vessels (García-Cáceres et al., 2016). The specific mechanism involves insulin signaling deficiency, which leads to mitochondrial fragmentation in astrocytes, followed by process atrophy, characterized by reductions in both the number and length of astrocytic processes. These morphological changes in astrocytes affect synaptic input and glucose metabolic signaling around POMC neurons, ultimately impairing the activation of POMC neurons (García-Cáceres et al., 2016). POMC neurons can sense glucose concentration and play an important role in maintaining glucose homeostasis. Parton et al. found that hypothalamic POMC neurons are a class of glucose-excited neurons that sense rising blood glucose levels through ATP-mediated closure of K_ATP channels (Parton et al., 2007). Under high-fat conditions, however, the excitatory response of POMC neurons to glucose is markedly attenuated, and this loss of function is associated with upregulation of mitochondrial uncoupling protein 2 (UCP2). UCP2 reduces the efficiency of ATP synthesis from glucose metabolism, thereby hindering the normal closure of K_ATP channels and consequently suppressing the sensitivity of POMC neurons to glucose (Parton et al., 2007). In summary, insulin acts on hypothalamic astrocytes to synergistically regulate glucose uptake and lactate utilization, while influencing glia–neuron structural and functional coupling. Together with neurons, this forms a glucose-sensing network that participates in the central nervous system’s regulation of systemic blood glucose homeostasis.

### 4.2 Leptin

Obesity is one of the major risk factors for type 2 diabetes. Persistent energy surplus and adipose tissue expansion trigger chronic low-grade inflammation, lipotoxicity, and changes in the adipokine profile, which impair glucose uptake in skeletal muscle and reduce hepatic insulin-mediated suppression of gluconeogenesis, resulting in systemic insulin resistance. Notably, obesity-related glucose metabolism disorders are not solely determined by peripheral organs: central metabolic regulatory networks, such as the hypothalamus, undergo remodeling in obesity through altered perception and output of peripheral nutritional signals. This creates a vicious cycle of peripheral insulin resistance—central dysregulation, which mutually reinforces each other. These mechanisms provide the physiological basis for adipokines (especially leptin) to link obesity status with glucose abnormalities.

Leptin, a protein hormone primarily secreted by white adipose tissue, is proportional to fat storage (Perakakis et al., 2021). Peripheral leptin must cross the blood–brain barrier to reach the central nervous system. The classical view holds that blood–brain barrier endothelial cells transport leptin from the blood into the brain, where the hypothalamus receives leptin signals and initiates responses such as appetite suppression (Hsuchou et al., 2009). Short-term high-fat diets impair hypothalamic glucose sensing, while supplementing leptin signaling partially corrects this sensory defect, thereby influencing peripheral glucose homeostasis. Evidence from insulin-deficient models demonstrates that continuous intracerebroventricular (ICV) leptin administration significantly improves (even nearly restores) glucose control and survival outcomes in insulin-deficient mice (Fujikawa et al., 2013). The key neural circuit involves the synergistic action of leptin receptors on hypothalamic GABAergic neurons and POMC neurons. Restoring LepR expression in these two neuron types alone suffices to mediate leptin’s core anti-diabetic effects in an “insulin-free” context, and this pathway operates independently of *β*-adrenergic receptors and direct insulin action. Mechanistically, leptin enhances peripheral glucose utilization through this hypothalamic pathway—particularly in brown adipose tissue (iBAT) and soleus muscle, while simultaneously improving hepatic metabolic status. Together, these actions constitute critical components for leptin’s hypoglycemic effects in peripheral tissues (Fujikawa et al., 2013).

Beyond neurons, the responsiveness of astrocytes to leptin signaling has provided new insights into the central regulation of peripheral metabolism. Studies have shown that leptin receptors (ObR) are widely expressed in hypothalamic astrocytes, and both ObR levels and GFAP expression are upregulated under diet-induced obesity (Hsuchou et al., 2009). However, this receptor upregulation does not signify enhanced leptin sensitivity. On the contrary, the authors proposed that the increase in astrocytic ObR expression participates in the mechanism of leptin resistance. This seemingly paradoxical phenomenon can be explained from two perspectives. First, at the molecular level, the upregulated ObR in astrocytes consists predominantly of the short isoform (ObRa), rather than ObRb, which is capable of activating the STAT3 signaling pathway to suppress food intake and reduce body weight (Hsuchou et al., 2009). Second, at the functional level, although leptin elicits a robust Ca^2+^ signal in primary hypothalamic astrocytes, this response undergoes rapid and substantial desensitization upon repeated stimulation, indicating that the signaling efficacy is impaired under sustained hyperleptinemia. Thus, the obesity-associated upregulation of astrocytic ObR and the Ca^2+^ signaling it mediates do not restore but rather remodel hypothalamic nutrient sensing and feeding circuitry, constituting a distinctive component of central leptin resistance (Hsuchou et al., 2009).

Conditional knockout of the LepR receptor specifically in ARH astrocytes directly alters astrocyte morphology, reducing the number and length of glial processes and decreasing neuronal soma envelopment. This is accompanied by increased synaptic input to the POMC/AgRP circuit and heightened synaptic event frequency, ultimately leading to weakened leptin-mediated appetite suppression, diminished POMC activation, and enhanced pro-feeding responses. This suggests that glial LepR signaling regulates feeding circuit plasticity and behavioral output through glia–neuron interfaces (Kim et al., 2014).

### 4.3 Ghrelin

Ghrelin is a peptide hormone primarily produced by the stomach that induces feeding behavior by activating ghrelin receptors in the central nervous system (Klok et al., 2007). It also serves as the endogenous ligand for the growth hormone secretagogue receptor (GHSR) (Kojima et al., 1999). Under fasting conditions, elevated circulating ghrelin levels activate AgRP and NPY neurons expressing GHSR in the ARC of the hypothalamus. These neurons project strongly to the PVH, accompanied by morphological remodeling of the ARH^AgRP/NPY^ → PVH pathway, thereby promoting foraging behavior to maintain energy and glucose homeostasis (Cabral et al., 2020).

Beyond direct neuronal effects, ghrelin participates in central energy metabolism regulation by modulating neuron–glia interactions. First, ghrelin at physiological concentrations induces significant intracellular Ca^2+^ responses in astrocytes (Marina et al., 2018), suggesting it can modulate astrocytic excitability and neurotransmitter release. At the metabolic sensing level, Fuente-Martín et al. found that acylated ghrelin reduces GLUT2 levels in hypothalamic astrocytes via GHSR (Fuente-Martín et al., 2016). Although this study did not directly investigate glucose sensing, based on the mechanism described in Section 1.2 above (Rogers et al., 2020), it is reasonable to infer that ghrelin-induced downregulation of GLUT2 would directly impair the ability of astrocytes to detect changes in extracellular glucose concentration. Specifically, GLUT2 reduction leads to an increased activation threshold of the PLC-calcium signaling pathway, thereby attenuating the CRR-triggering signal to hindbrain catecholaminergic neurons. Moreover, acylated ghrelin can also maintain extracellular glutamate homeostasis by increasing the glutamate clearance capacity of astrocytes, thereby reducing the risk of excitotoxicity to adjacent neurons (Fuente-Martín et al., 2016). In other words, ghrelin not only directly acts on pro-feeding neurons like AgRP/NPY but also indirectly modulates neuronal excitability by regulating the capacity of astrocytes to maintain synaptic glutamate homeostasis. Furthermore, astrocytes can act as negative feedback brakes to counter-regulate ghrelin’s drive for feeding. Existing models demonstrate that ghrelin enhances AgRP neuron activity by promoting presynaptic glutamate release (Abizaid et al., 2006), while simultaneously activating astrocytic glutamate receptors (Biber et al., 1999) and triggering ATP release (Bal-Price et al., 2002). ATP is further degraded into adenosine (Dunwiddie et al., 1997), which inhibits AgRP neuron firing via A1 receptors, thereby suppressing ghrelin-induced feeding (Yang et al., 2015). Astrocytes regulate synaptic neurotransmission to confine hunger drives within controlled limits, enabling the central circuitry to dynamically balance pro-feeding and glucose conservation outputs. Collectively, ghrelin’s regulation of astrocytes represents a crucial component of neuron–glia cooperation, offering new insights into its role in glucose homeostasis.

### 4.4 Estrogen

Estrogen, a crucial sex hormone, not only plays a key role in the reproductive system but also regulates energy metabolism and blood glucose homeostasis through the CNS. Extensive clinical and animal studies have shown that estrogen (primarily 17β-estradiol) improves glucose handling and enhances insulin sensitivity. Conversely, estrogen deficiency states—such as menopause or oophorectomy—often result in weight gain, insulin resistance, and impaired glucose metabolism (Esmailidehaj et al., 2020). These findings support the notion that central estrogen signaling contributes to the regulation of glucose homeostasis, though the key cellular targets and circuit mechanisms remain to be further elucidated. At the cellular level, estrogen signaling does not act exclusively on neurons. Astrocytes extensively express estrogen receptor alpha (ERα), estrogen receptor beta (ERβ), and G protein-coupled estrogen receptor 1(GPER1) (Fuente-Martin et al., 2013), which provides the anatomical and molecular basis for estrogen-mediated modulation of neural circuit function through the astrocyte-neuron cooperative unit. Consistent with this, studies demonstrate that estrogen rapidly modulates astrocytic excitability and glial neurotransmitter release. Local application of 17β-estradiol(E2)increases elevated intracellular Ca^2+^ signaling in astrocytes via the phospholipase C (PLC)/inositol trisphosphate (IP3) pathway, promoting glutamate release that generates slow inward currents (SICs) in adjacent neurons. This process involves glutamate released by astrocytes activating extrasynaptic NMDA receptors on neurons, forming an estrogen-mediated signaling pathway that acts through astrocytes and ultimately targets neurons (Goenaga et al., 2025).

Beyond signal transduction, estrogen has been shown to enhance astrocytes’ capacity for clearing glutamate from the microenvironment and maintaining its homeostasis. 17β-estradiol (E2) significantly elevates mRNA and protein levels of the primary glutamate transporters GLT-1 (EAAT2) and GLAST (EAAT1) in astrocytes, along with their glutamate uptake activity (Karki et al., 2014). This effect is not solely dependent on classical nuclear ERα/ERβ pathways but also involves GPER1, which promotes GLAST and GLT-1 upregulation, thereby preventing extrasynaptic glutamate accumulation and associated excitotoxicity. Therefore, existing evidence supports that estrogen modulates astrocyte function through multiple pathways, including Ca^2+^ signaling, glial glutamate release, and glutamate transport clearance. This provides a foundational framework for mechanisms that remodel the extracellular glutamate microenvironment and consequently influence neuronal activity.

However, it is important to emphasize that current evidence supporting the direct regulation of blood glucose by estrogen via hypothalamic astrocyte-neuron crosstalk is still largely indirect. First, direct evidence of estrogen regulating astrocytic Ca^2+^ dynamics and glutamate transport mainly originates from hippocampal/cortical or *in vitro* systems, with research endpoints primarily focused on neuroprotection and synaptic plasticity. Second, while studies on hypothalamic astrocytes influencing systemic glucose metabolism have established a causal link between the glial microenvironment and metabolic phenotypes, direct evidence is still lacking. This includes the absence of systematic genetic manipulation of astrocytic ERα/ERβ/GPER1 signaling axes within the same animal model and hypothalamic nucleus, along with assessment of glucose homeostasis and hypoglycemic counter-regulation phenotypes. Therefore, future studies may employ astrocyte-specific genetic strategies (e.g., GFAP-Cre or ALDH1L1-Cre-mediated conditional knockout and knockin of ERα or GPER1) combined with glucose homeostasis and hypoglycemia counterregulation analyses to test the causal validity of the proposed working model and clarify its contribution within the hypothalamic glucose control network.

### 4.5 Glucagon-like peptide-1

Glucagon-like peptides (GLPs) are a family of peptide hormones produced by enteroendocrine L-cells in the intestine and specific neurons in the central nervous system, primarily comprising GLP-1 and GLP-2 (Calsolaro and Edison, 2015). GLP-1 can inhibit gastric emptying, reduce food intake, and exhibit potent insulinotropic effects, making it a key therapeutic target for metabolic disorders such as type 2 diabetes and obesity (Drucker, 2018). In the central nervous system, GLP-1 signaling originates predominantly from preproglucagon (PPG)-expressing neurons located in the nucleus of the solitary tract (NTS) (Larsen et al., 1997).

Astrocytic glucagon-like peptide-1 receptor (GLP-1R) signaling plays a central regulatory role in energy metabolism. Using fluorescently labeled exendin-4, Reiner et al. demonstrated that GLP-1R is not only present on NTS neurons but also localizes to astrocytes within this brain region (Reiner et al., 2016). Their experimental findings revealed that activation of astrocytic GLP-1R significantly elevates intracellular cAMP and Ca^2+^ levels in astrocytes, and pharmacological inhibition of NTS astrocytes attenuates the anorectic and body weight-suppressive effects of intra-NTS GLP-1R activation (Reiner et al., 2016). Based on these observations, the authors propose that GLP-1R activation may downregulate the expression of glutamate transporters (GLT-1 and GLAST), thereby increasing synaptic glutamate levels to enhance NTS-mediated satiety signaling and ultimately suppress food intake (Reiner et al., 2016).

Furthermore, Timper et al. systematically elucidated the complex physiological functions of astrocytic GLP-1R signaling (Timper et al., 2020). In cultured hypothalamic astrocytes, acute GLP-1 treatment promoted fatty acid *β*-oxidation while simultaneously suppressing glucose uptake, indicating that physiological activation of GLP-1R drives a metabolic fuel switch from glucose utilization toward fatty acid oxidation. Conversely, specific deletion of GLP-1R in hypothalamic astrocytes impaired mitochondrial integrity, as evidenced by decreased oxidative phosphorylation capacity and the loss of adaptive mitochondrial morphological changes in response to acute glucose loading. This was accompanied by elevated expression of fibroblast growth factor 21 (FGF21), a downstream effector of the integrated stress response. FGF21 improved systemic glucose metabolism and enhanced memory formation through modulation of dopaminergic neuron activity, an effect interpreted as a compensatory stress response. Notably, in the context of GLP-1R deficiency, astrocytes also exhibited increased phosphorylation of GLUT1 at Ser-226, leading to enhanced glucose uptake and glycolytic flux to compensate for impaired oxidative phosphorylation. These cell-autonomous compensatory adaptations, via FGF21-mediated intercellular signaling to other cell types and peripheral organs, resulted in improved insulin sensitivity in the liver and adipose tissue, thereby enhancing systemic glucose homeostasis.

## 5 Hypothalamic astrocytes in type 2 diabetes

However, this astrocyte-mediated regulatory system is disrupted during metabolic imbalance. Prolonged exposure to high glucose and high fat levels in type 2 diabetes and its precursor stages impairs brain homeostasis, with metabolic centers like the hypothalamus often exhibiting the earliest signs of neuroinflammation and glial responses. Astrocytes undergo reactive remodeling during this process, altering inflammatory mediator and purinergic signaling outputs. Consequently, this section focuses on the impact of astrocytic alternations in type 2 diabetes on neural circuits and systemic metabolism.

At the morphological level, chronic metabolic stress (e.g., high-fat diet, HFD) induces significant reactive gliosis. In the ARH and ME regions, astrocytic marker GFAP expression is markedly upregulated, with thickened cell processes forming dense fibrillar networks (Horvath et al., 2010). Ultrastructural imaging reveal that proliferating astrocytic processes not only alter synaptic input patterns to POMC neurons (e.g., reduced inhibitory synapses) but, more critically, envelop the somata of POMC and NPY neurons. This leads to physical isolation of neurons from the local microvasculature. This anatomical barrier formation may impede neuronal perception of circulating metabolic signals (e.g., leptin, insulin), thereby exacerbating metabolic dysregulation.

Clinical imaging evidence further confirms the association between these pathological alterations and glucose homeostasis. Quantitative MRI studies reveal that compared to obese individuals with normal glucose tolerance (NGT), the degree of gliosis measured by T2 relaxation time is significantly elevated in the hypothalamic mediobasal region (MBH) of patients with impaired glucose tolerance (IGT) and T2DM (Rosenbaum et al., 2022). This gliosis shows a significant negative correlation with *β*-cell function and insulin sensitivity. This glial proliferation in the MBH is believed to disrupt glia–neuron interactions (e.g., the arcuate nucleus POMC neuronal pathway), leading to central glucose dysregulation. Collectively, these findings suggest that morphological and functional remodeling of astrocytes represents a key pathological link connecting obesity to central glucose dysregulation. Although the cellular mechanisms underlying these discoveries remain poorly understood due to limitations in *in vivo* imaging resolution, substantial evidence now supports a pivotal role for astrocytes and their interactions with neurons in the progression of type 2 diabetes. Further investigation into the detailed mechanisms requires future exploration.

Neuroinflammation is also a key pathological feature of T2DM, with the hypothalamic inflammation-insulin resistance axis considered a central mechanism driving T2DM progression (Zhang et al., 2008). Under metabolic stress, glial cells in the hypothalamus—including astrocytes—can shift from a homeostatic support phenotype to a reactive state, thereby initiating and amplifying inflammatory signaling. Under conditions of high glucose exposure and fluctuating glucose concentrations, hypothalamic astrocytes are prone to reactive activation: enhanced NF-κB signaling with bursts of ROS/RNS initiates and amplifies glial inflammatory responses (Quincozes-Santos et al., 2017). This transformation directly results in the upregulation of proinflammatory factors such as TNF-*α*, IL-1β, and IL-6, coupled with decreased levels of the anti-inflammatory factor IL-10. This transforms astrocytes from homeostatic supporters into inflammatory amplifiers, persistently exacerbating the local inflammatory microenvironment in the hypothalamus.

High-fat diet-associated innate immune recognition can also serve as an upstream trigger, further promoting the initiation and maintenance of hypothalamic glial inflammatory responses. Specifically, the Toll-like receptor (TLR) signaling axis and its downstream myeloid differentiation primary response 88 (MyD88) pathway in hypothalamic astrocytes are implicated in regulating reactive gliosis and hypothalamic inflammation. MyD88 serves as a critical adaptor protein in TLR signaling; under inflammatory conditions, TLR4 activation significantly initiates downstream MyD88-dependent pathways. Compared to controls, HFD-induced reactive gliosis was markedly reduced in MyD88-deficient mutant mice. Furthermore, astrocyte-specific MyD88 deficiency reduced the intensity of intracellular signaling activation induced by HFD, decreased hypothalamic inflammatory cytokine expression, and maintained leptin-induced signal transducer and activator of transcription 3 (STAT3) phosphorylation levels, thereby improving leptin resistance (Jin et al., 2020). These findings indicate that TLR–MyD88 signaling within astrocytes plays a crucial mediating role in HFD-induced reactive gliosis and hypothalamic inflammatory amplification, and is associated with leptin resistance.

It should be noted that although TLR4 activation leads to MyD88-dependent proinflammatory cytokine release from astrocytes, the specific molecular mechanisms remain incompletely elucidated (de Git and Adan, 2015). Research by Birla et al. provides important clues. They discovered in spinal cord astrocytes that TLR4 activation enhances IL-6 and TNF-*α* production during inflammation by upregulating calcium release-activated calcium channels (CRAC) (Birla et al., 2022). However, this evidence primarily originates from spinal cord astrocytes. Whether hypothalamic astrocytes similarly exhibit the TLR4 → CRAC → proinflammatory factors coupling mechanism requires further validation.

Furthermore, *in vitro* experiments indicated that saturated fatty acids (SFAs) and pro-inflammatory cytokines can directly activate cultured astrocytes, thereby inducing the release of pro-inflammatory cytokines (Gupta et al., 2012). Specifically, SFAs directly activate the Toll-like receptor 4 (TLR4) on astrocytes, thereby upregulating the expression of pro-inflammatory factors such as IL-6, IL-1*β*, and TNF-*α*. Subsequently, these cytokines further act on cytokine receptors in POMC and AgRP neurons, inducing and amplifying IKKβ (inhibitor of nuclear factor kappa B kinase subunit β)/NF-κB signaling within neurons (de Git and Adan, 2015). Moreover, the IKKβ/NF-κB pathway not only serves as a central hub in the inflammatory transcription program but also directly interferes with insulin and leptin signaling. This pathway induces serine phosphorylation of insulin receptor substrate (IRS), a key shared effector molecule for insulin and leptin signaling, thereby inhibiting insulin and leptin signaling and disrupting energy homeostasis regulation (Sergi and Williams, 2020). Moreover, the IKKβ/NF-κB signaling pathway upregulates the expression of cytokine-regulated inhibitor 3 (SOCS3), which directly interferes with the leptin receptor and inhibits leptin-induced STAT3 activation, further exacerbating leptin resistance. Thus, the SFAs-triggered astrocytic TLR4 inflammatory output and neuronal IKKβ/NF-κB signaling abnormalities are mechanistically interconnected, jointly constituting the critical pathway for the conversion of hypothalamic inflammation into hormone resistance and metabolic disorders under the HFD context.

## 6 Astrocytes beyond the hypothalamus

The role of astrocytes in glucose homeostasis extends beyond the hypothalamus. Increasing evidence indicates that astrocytes located outside the hypothalamus also participate in glucose sensing and regulation, with the nucleus of the NTS in the dorsal medulla being one of the key brain regions. The NTS serves as a vital integrative hub for multiple autonomic functions. It primarily receives signals from organs such as the lungs, gastrointestinal tract, and heart via bilateral vagus nerve afferent fibers. After integration, it projects information to relevant brainstem and forebrain regions, thereby participating in neuroendocrine and autonomic regulation (Andresen and Kunze, 1994).

In glucose homeostasis regulation, particularly during the counter-regulatory response (CRR) to hypoglycemia, astrocytes within the NTS play a central role. Studies demonstrated that astrocytes in the posterior NTS exhibited robust intracellular calcium signaling in response to glucose deprivation (e.g., low-sugar stimulation or 2-deoxyglucose, 2-DG). The specific mechanism involved GLUT2 glucose transporters expressed by astrocytes, which bound to the PLC pathway to trigger calcium release from the endoplasmic reticulum (ER) (Leloup et al., 1994). This caused a significant increase in cytoplasmic Ca^2+^, with both the response amplitude and kinetic characteristics (peak and rise rate) being stronger than those in adjacent neurons. More importantly, following tetrodotoxin (TTX)-induced block of neuronal action potentials, astrocytes retained their low-glucose Ca^2+^ response. This suggests glucose sensing is independent of local neuronal firing activity and likely represents a relatively direct monitoring mechanism for glucose availability (McDougal et al., 2013). At the circuit level, NST astrocytes may drive CRR by influencing catecholaminergic (CA) neurons. The NST and catecholaminergic (CA) neurons in the ventrolateral medulla (VLM) are considered key pathways for CRR output (Ritter et al., 2011), regulating hormone secretion (epinephrine, corticosterone) and hepatic glycogenolysis. In a 2-deoxy-D-glucose (2-DG)-induced intracellular glucose depletion model, 90% of CA neurons were activated. However, when astrocyte metabolism was inhibited with fluorocitrate (FC), CA neuron responses to low glucose stimuli were significantly attenuated or even abolished (Hermann et al., 2014). Furthermore, the Ca^2+^ response of CA neurons to 2-DG disappeared after administration of DPCPX (an A1 adenosine receptor antagonist) (Rogers et al., 2016). These findings support that astrocytes occupy a relatively upstream position in the CRR response pathway. Under hypoglycemic conditions, they release substances such as ATP/adenosine to modulate the excitability and output of downstream CA neurons, thereby influencing CRR intensity.

Beyond hypoglycemic counterregulation, NTS astrocytes also mediate trauma-induced hyperglycemic responses. Thrombin levels significantly increase during severe trauma such as hemorrhage or burns. Previous studies suggested thrombin directly acts on protease-activated receptors (PARs) on NTS astrocytic membranes, inducing adenosine release. This adenosine subsequently activated adenosine A1 receptors on adjacent neurons, leading to elevated blood glucose (Rogers et al., 2020). Thus, NTS astrocytes can be regarded as an integrated glial sensor: they perceive energy substrate availability (hypoglycemia) while converting trauma-related humoral signals (thrombin) into purinergic glial transmission, subsequently influencing systemic glucose metabolism via autonomic neural circuits. Based on this, targeting purinergic glial transmission in NTS astrocytes or its metabolic coupling mechanisms may offer novel intervention strategies for post-traumatic hyperglycemia.

## 7 Concluding remarks and future perspectives

In recent years, substantial progress has been made in understanding how astrocytes—particularly those in the hypothalamus—contribute to blood glucose regulation. Astrocytes are increasingly recognized as active participants in the central network governing glucose homeostasis, rather than as passive metabolic support cells. This review summarizes how astrocytes sense glucose, coordinate with neurons and associated circuits, and integrate peripheral hormonal and nutrient cues to influence counter-regulatory responses, autonomic output, and peripheral glucose production. The traditional neuron-centric paradigm of glucose regulation should shift toward a neuron–glia cooperative framework. Future in-depth analyses of astrocyte heterogeneity, plasticity, and intercellular signal coupling will help elucidate central mechanisms underlying diabetes and impaired awareness of hypoglycaemia, and inspire new intervention strategies targeting central metabolic control.

Despite rapid advances in recent years, several key questions regarding astrocyte-mediated regulation of glucose homeostasis remain unresolved. The hypothalamus is not composed of a single neuronal type; rather, it contains multiple neuronal subpopulations with distinct molecular signatures and physiological functions. Current studies largely emphasize astrocyte coordination with entire brain regions, whereas investigations focusing on astrocyte interactions with specific neuronal subpopulations within hypothalamic nuclei are relatively limited. Specific neuronal subpopulations within these regions can communicate directly with peripheral target organs (e.g., liver and pancreas) via autonomic pathways, thereby playing pivotal roles in regulating glucose production and the secretion of insulin and glucagon. Accordingly, future work could integrate single-cell/single-nucleus transcriptomics with spatial transcriptomics to first identify candidate astrocyte–neuronal subpopulation interactions, followed by the use of astrocyte-specific Cre systems with viral vectors, optogenetics, and chemogenetics to manipulate astrocytes within defined circuits while assessing peripheral metabolic phenotypes. Integrating these approaches with systems-level metabolic phenotyping may help clarify the mechanisms by which astrocytes coordinate central–peripheral control of glucose homeostasis.

From a translational perspective, astrocyte dysfunction may represent a previously underappreciated but important pathological contributor in metabolic diseases. Hypothalamic astrocyte–mediated neuroinflammatory responses are believed to participate in early stages of diet-induced obesity and central insulin resistance. Chronic overnutrition and sustained hyperglycemia may induce astrocytic morphological remodeling, metabolic reprogramming, and proinflammatory cytokine production. These changes can reduce astrocytic responsiveness to metabolic hormones such as insulin and leptin, thereby disrupting central glucose homeostasis. Therefore, interventions targeting astrocyte-specific signaling pathways or inflammatory mediators may offer new therapeutic avenues for obesity and type 2 diabetes. For example, modulating NF-κB–associated inflammatory signaling and restoring the IKKβ–SOCS3 signaling pathway may alleviate central inflammation and enhance metabolic hormone sensitivity.
